# Mediating role of social support in relations between psychological capital and subjective well-being among IDF soldiers during conflict: insights from the 2023 Gaza war

**DOI:** 10.1186/s13584-025-00713-4

**Published:** 2025-08-21

**Authors:** Batel Hazan-Liran, Ofra Walter

**Affiliations:** Faculty of Education, Tel Hai Academic College, Kiryat Shmona, Israel

**Keywords:** Psychological capital, Social support, Subjective well-being, Israel defense forces, Resilience, Combat stress, 2023 gaza war, Psychological resilience

## Abstract

**Background:**

The study explored the relations between psychological capital, social support, and subjective well-being among Israel Defense Forces soldiers, focusing on differences between active-duty and reserve soldiers, as well as pre-war and wartime conditions.

**Methods:**

The sample comprised 233 soldiers, divided into three groups based on their service conditions: active-duty soldiers before the war, active-duty soldiers during the war, and reserve soldiers during the war. Four questionnaires were administered: Demographic Questionnaire, Psychological Capital Questionnaire, Social Support Questionnaire, and Subjective Well-Being Questionnaire.

**Results:**

The findings revealed significant positive correlations between psychological capital, social support, and subjective well-being, with social support mediating relations. While psychological capital remained stable across various service conditions, social support and subjective well-being were more pronounced among soldiers serving during wartime, underscoring the vital role of interpersonal connections in high-stress environments.

**Conclusion:**

The results suggest psychological capital may function as a psychological buffer, positively associated with resilience and well-being. Moreover, the mediation effect of social support highlights the importance of social networks in sustaining psychological resilience and mitigating the adverse effects of combat stress. While these are established constructs, the study embeds them within a real-time war context, offering rare insight into how they operate under acute national trauma. Given the cross-sectional design, findings should be interpreted as associations rather than definitive causal relationships.

## Background

The Israel Defense Force (IDF) plays a pivotal role in ensuring the security of Israel, with soldiers facing unique psychological and social challenges during their service [1]. Military service, especially in high-stress environments, can have significant effects on soldiers’ well-being, with negative mental health outcomes, such as anxiety, depression, and post-traumatic stress disorder (PTSD) [2, 3]. While the IDF has implemented various psychological support programs to mitigate these effects [4], understanding the underlying factors that contribute to resilience and subjective well-being (SWB) in soldiers remains crucial for improving their mental health and operational effectiveness [1, 5].

One such factor is psychological capital (PsyCap). It includes resilience, optimism, hope, and self-efficacy and has been linked to better coping mechanisms and mental health outcomes in military contexts [6, 7]. PsyCap plays a key role in fostering a motivational mindset that helps individuals navigate adversity, and it has been shown to influence soldiers’ ability to adapt to the stresses of military life [8]. Social support has been identified as another important buffer against the negative effects of stress, promoting resilience and enhancing overall well-being, including in military contexts [9, 10]. The relationship between PsyCap and social support is likely to be reciprocal, with higher levels of psychological resources leading to stronger social networks, which, in turn, can enhance soldiers’ resilience and subjective well-being (SWB) [11, 12].

The study was conducted in the context of Israel’s ongoing conflict in the Gaza Strip, which has significantly impacted civilians [13]. A pivotal escalation occurred on October 7, 2023, when Hamas-led militants launched a large-scale attack, resulting in substantial civilian and military casualties [14–16]. The assault involved coordinated invasions, rocket barrages, and attacks on civilian sites, including a music festival near Re’im. The IDF played a central role in responding to the attack, with varying effects on different groups of soldiers. Active-duty soldiers before the war were engaged in routine security operations along the Gaza border, monitoring threats and maintaining defenses. Following the attack, active-duty soldiers were rapidly deployed for intense urban combat, hostage rescues, and retaliatory strikes, leading to extreme psychological and operational stress [16]. The conflict also prompted the largest mobilization of IDF reserve soldiers in recent history, with over 300,000 called up. Reservists transitioned abruptly from civilian life to combat roles, facing high stress and logistical challenges due to limited preparation time [17]. These differences in service status shaped the soldiers’ experiences, levels of preparedness, and the war’s psychological impact.

Recent studies have begun to examine how Israeli civilians experienced distress and relied on coping resources during the Iron Swords War, underscoring the urgency of investigating psychological mechanisms in real-time wartime contexts [13, 16]. We added to the literature by exploring how PsyCap and social support interact to shape soldiers’ subjective well-being during real-time active conflict, offering novel insights into their role under acute national threat. More specifically, we examined the interplay between PsyCap, social support, and SWB among IDF soldiers, focusing on how these factors varied between active-duty soldiers before and during the recent conflict escalation. We expanded the study by considering reservists called up for duty. By exploring whether social support mediates the relationship between PsyCap and SWB, the research contributes valuable insights into the psychological mechanisms that help soldiers cope with the demands of military service [18, 19]. Importantly, the study extends previous work by situating these constructs within a unique high-threat, real-world conflict. It contributes to theory by empirically testing PsyCap and social support dynamics in the wartime context and considering their interaction in active-duty versus reserve soldiers. The findings advance the understanding of psychological resilience in extreme operational environments [1, 16, 20–22].

### IDF: history and structure

The Israel Defense Forces (IDF), established in 1948, is a central institution that has played a critical role in national defense while also shaping Israel’s identity and social cohesion [1, 23]. Its organizational structure and doctrine have evolved to meet shifting threats from both state and non-state actors [24]. Comprising the Ground Forces, Air Force, Navy, and intelligence and cyber units [1], the IDF has increasingly prioritized specialized operations over traditional warfare, reflected in the expansion of cyber and special forces and the downsizing of conventional combat units [4, 5].

Military service, particularly within the IDF, can significantly impact soldiers’ personal well-being. On the one hand, it contributes to a sense of belonging, camaraderie, and social engagement, fostering a strong sense of identity and purpose [2, 25, 26]. On the other hand, the demanding nature of military life, including exposure to combat situations, high levels of stress, and the pressure to perform, can lead to mental health challenges such as anxiety, depression, and PTSD [1, 3]. These emotional and psychological burdens can affect soldiers’ overall quality of life, both during and after their service [2, 26]. Consequently, the IDF has increasingly focused on providing psychological support and resilience-building programs [4] to maintain soldiers’ operational effectiveness and overall well-being [1, 5].

Research indicates mental resilience is linked with good mental health, while reduced resilience is connected with an increased risk for disturbances in mental health [3, 24]. However, some soldiers are reluctant to seek mental health assistance, often due to concerns about perceived weakness or potential impact on their military careers [3]. Addressing these barriers is essential to foster a supportive environment where soldiers feel safe to seek help. For example, the IDF has established dedicated facilities like the National Center for Mental Health and Resilience. This center provides vital therapy for traumatized, active-duty soldiers, addressing issues such as PTSD and trauma. Additionally, initiatives like “Shields of Resilience” offer combat soldiers tools and support to safely transition from the battlefield, aiming to prevent PTSD and promote mental well-being [1]. Despite these efforts, challenges remain.

### PsyCap and soldiers

PsyCap is a concept originating in positive psychology, a field aiming to enhance individual well-being by fostering psychological health [27]. PsyCap represents a positive psychological state made up of four dimensions: self-efficacy, hope, optimism, and resilience [8]. These components work together to foster a motivational mindset that supports success even in the face of adversity [6], including during and after military service [25].

Self-efficacy refers to individuals’ belief in their capabilities to perform tasks effectively and is rooted in motivation, cognitive resources, and past experiences [28]. Hope is the belief in one’s ability to generate multiple pathways towards goal achievement, maintaining motivation and perseverance in the pursuit of these goals [29]. Optimism reflects a general tendency to expect positive outcomes and interpret challenges as opportunities for growth [30]. Finally, resilience is the capacity to recover and adapt following setbacks or distress, fostering long-term success [8]. Each dimension contributes to the overall development of PsyCap, helping individuals navigate challenges, persist through adversity, and maintain a sense of motivation and belief in their goals. The combination of these four elements results in a unified motivational personality construct that can be developed over time, enabling individuals to cope more effectively with stress and uncertainty [8, 31].

A growing body of research underscores the significance of PsyCap in various domains, including in military contexts. Studies conducted on US military personnel have demonstrated that high levels of PsyCap are associated with reduced risks of mental health issues such as PTSD, anxiety, and depression. Soldiers exhibiting higher levels of PsyCap are also less likely to develop substance use disorders [7, 32].

In addition to its association with mental health outcomes, PsyCap has been shown to positively influence SWB and job satisfaction in workplace settings [33, 34]. Military studies have found PsyCap significantly contributes to soldiers’ overall well-being and satisfaction [35]. In fact, recognizing the value of PsyCap in improving resilience and coping mechanisms, the US military implemented a resilience-building program in 2008 to enhance PsyCap components among soldiers and their families stationed at military bases [35].

Recent studies on the military environment highlight the critical interplay between the core components of PsyCap. Researchers have shown these factors are deeply interconnected, with resilience serving as a stabilizing force that amplifies the impact of hope and optimism, thereby fostering an individual’s sense of self-efficacy [36]. This suggests PsyCap can be cultivated not only to improve mental health but also to bolster overall performance, especially in high-stress environments like the military. Newer findings from military studies emphasize the importance of developing PsyCap as part of training programs to enhance soldiers’ ability to cope with the rigors of military life, including combat situations. These programs aim to foster psychological flexibility, enabling soldiers to adjust their mental and emotional responses to stressful events and recover more effectively [37].

### SWB and soldiers

SWB refers to individuals’ self-assessment of their overall life satisfaction and emotional experiences across various life domains. The concept of SWB is typically broken down into two major components. The first component, affective well-being, captures the presence of positive emotions, such as happiness and joy, and the absence of negative emotions, such as sadness, anger, or anxiety. The second component, cognitive well-being, pertains to the overall life satisfaction that individuals perceive in various aspects of their lives, such as their professional life, social relationships, and general fulfillment [38]. These evaluations take into account individuals’ current state, but also how they reflect on their life as a whole, thus spanning a temporal continuum from present to past [39]. Examining these two components allows researchers to understand how life satisfaction and emotional states are intricately intertwined, offering a multifaceted view of a person’s subjective experience.

Predictors of SWB include an individual’s life circumstances, genetic predispositions, personality traits, and cultural or demographic factors [39, 40]. These factors combine in unique ways to influence the SWB that individuals report. High levels of SWB are linked to a variety of positive outcomes. For example, individuals with higher SWB report better health outcomes, higher income, greater job satisfaction, stronger social relationships, and an overall sense of contribution to society [41, 42]. Consequently, SWB is not only an indicator of personal fulfillment but also a predictor of broader societal participation and success in multiple life domains.

Military service, especially combat experience, has been shown to have a significantly negative impact on soldiers’ SWB [43, 44]. Numerous studies have found exposure to combat leads to a sharp decline in SWB [45]. This decrease is particularly evident post-combat, as soldiers often continue to face profound psychological challenges, such as PTSD and depression [7, 32, 46]. In the United States, for instance, studies show veterans who have served in combat zones commit more crimes and report higher rates of violence and suicide than their non-combatant counterparts [47]. A survey conducted among US soldiers who served in Iraq or Afghanistan found 26% of them reported lower SWB upon returning, with many also exhibiting increased tendencies toward depression, suicidal thoughts, and substance abuse [48].

### Social support and soldiers

Social support refers to interactions between individuals that foster the belief that one is loved, valued, and cared for. It serves as a protective factor, particularly in stressful situations, by enhancing an individual’s resilience to stress, reducing engagement in risky behaviors, and preventing negative self-evaluations [9, 49]. Social support comprises not only emotional support, which provides a sense of being valued, but also tangible assistance, such as financial aid, and informational support, which encompasses advice, guidance, and problem-solving [50]. Such support plays a critical role in promoting positive self-esteem, self-efficacy, and overall well-being [51].

Some studies have suggested strong interpersonal relationships can act as a buffer against the negative effects of military service by bolstering SWB [10]. Soldiers who report strong connections with their intimate partners and close bonds with peers in their military units tend to exhibit higher levels of SWB, as social support plays a crucial role in mitigating the emotional toll of military service [45]. Soldiers with robust relationships have better emotional outcomes and are less likely to experience severe mental health issues post-service [52]. Those who feel supported by their social networks tend to exhibit higher psychological resilience, leading to more favorable mental health outcomes and greater overall life satisfaction [53, 54].

The enlistment and recruitment process in Israel is mandatory and necessitates a transition from a familiar environment to a highly structured military setting, a process that often triggers significant stress and psychological challenges [55]. The transition can lead to heightened psychological distress, which, in some cases, may contribute to substance abuse or depression [10]. Social support has been identified as a key factor in mitigating these effects, protecting individuals from stress and psychological difficulties [18]. More broadly, social support can enhance resilience to stress and reduce the risk of trauma-related psychopathology, underscoring its importance as a protective factor in high-stress environments [9].

Research on soldiers shows social support mitigates negative psychological outcomes in the context of combat [56]. Specifically, the role of family support has been highlighted as crucial for soldiers’ ability to cope with stressors in both combat situations and post-combat reintegration into civilian life [10]. Supportive family structures help foster resilience, which in turn, acts as a protective factor against the development of psychopathological issues following combat exposure [57].

Furthermore, studies have shown that social support contributes to the development of the components of PsyCap, further enhancing the ability to cope with adversity [11, 58]. In addition to its effects on PsyCap, social support has been linked to improved well-being in a broader sense. Research indicates social support serves as a valuable resource for reducing stress and burnout, while also contributing to greater SWB [19]. In fact, multiple studies have established social support as one of the strongest predictors of SWB [12, 59]. Consequently, the extent to which individuals receive social support from their immediate social networks plays a critical role in determining their overall sense of well-being [60].

One of the aims of the present study was to clarify whether social support mediates the relationship between PsyCap and SWB. PsyCap plays a crucial role in enhancing social support, particularly in high-stress environments like military settings [11, 43]. When soldiers possess high levels of PsyCap, they are more likely to engage in positive social interactions, seek support when needed, and foster stronger interpersonal connections [18, 19]. This enhanced social support, in turn, acts as a protective factor, buffering against stress and improving overall well-being [25, 59]. The reciprocal relationship between PsyCap and social support may create a virtuous cycle in which psychological resources empower individuals to build supportive networks, and these networks, in turn, further enhance their psychological resilience and personal well-being [10, 12]. This dynamic may significantly contribute to soldiers’ ability to cope with the challenges of military life, including combat stress and reintegration into civilian life, fostering greater SWB and better mental health.

### The present study

We examined the relationships between PsyCap, social support, and SWB among IDF soldiers in the context of the 2023–2024 Iron Swords war in Gaza, comparing three samples: a group of active-duty IDF soldiers who had been part of a previous study before the war, a group of active-duty soldiers who participated in the study during the war, and a group of reserve service soldiers who participated during the war. Based on the literature, we formulated the following research questions and hypotheses:

RQ1: Will there be positive correlations between PsyCap, social support, and SWB, both in active-duty soldiers in general and in the two groups: active-duty soldiers participating in the study before the war and active-duty soldiers participating during the war?

H1: There will be significant positive correlations between PsyCap, social support, and SWB in active-duty soldiers in general and in both groups: active-duty soldiers participating in the study before the war and those participating during the war.

RQ2: Will there be differences between active-duty soldiers participating in the study before the war and those participating during the war in their levels of PsyCap, social support, and SWB?

H2: Active-duty soldiers participating in the study during the war will exhibit higher levels of social support and SWB than active-duty soldiers participating before the war, but there will be uniform levels of PsyCap in both groups.

RQ3: Will social support mediate the relationship between PsyCap and SWB among active-duty soldiers participating in the study before and during the war?

H3: Higher levels of PsyCap will be associated with higher levels of social support, which in turn, will lead to greater SWB in both active-duty groups.

RQ4: Will there be positive correlations between PsyCap, social support, and SWB in both active-duty soldiers and reserve service soldiers during the war?

H4: There will be significant positive correlations in the overall sample and within both groups: active-duty soldiers and reserve service soldiers during the war.

RQ5: Will there be differences between active-duty soldiers and reserve service soldiers in their levels of PsyCap, social support, and SWB during the war?

H5: Reserve service soldiers will have higher levels of PsyCap, social support, and SWB than active-duty soldiers during the war.

RQ6: Will social support mediate the relationship between PsyCap and SWB in active-duty and reserve service soldiers during the war?

H6: Higher levels of PsyCap will be associated with higher levels of social support, which will lead to greater SWB in both active-duty and reserve service soldiers during the war.

## Method

### Participants

Participants were recruited using convenience sampling within IDF units. Participation was facilitated by army mental health officers and commanders who distributed the survey link during periods of operational availability. Although this method allowed timely access to three distinct groups, it may limit the representativeness of the sample.

A sample of 233 soldiers was divided into three groups based on their service conditions: active-duty soldiers before the war, active-duty soldiers during the war, and reserve soldiers during the war. Since the IDF has a male majority, the sample included 59% male soldiers and 41% female soldiers, with ages ranging from 18 to 60 years and a mean age of 27 (SD = 6.5). The soldiers’ service periods varied from 1 to 80 months, with an overall mean of 22 months (SD = 14.2). The group of active-duty soldiers before the war (*n* = 98) was predominantly male (59%), with an age range of 18 to 27 years (mean age of 21). In terms of service roles, 40% were combat soldiers, 31% were combat support soldiers, and 29% were combat service support soldiers. The service period for this group ranged from 10 to 80 months, with a mean of 22 months. The active-duty soldiers during the war (*n* = 76) included 49% males and 51% females, with an age range of 18 to 27 years (mean age of 21). In this group, 18% were combat soldiers, 21% were combat support soldiers, and 61% were combat service support soldiers, with service periods ranging from 10 to 68 months (mean of 32 months). Finally, the reserve soldiers during the war (*n* = 59) were mostly male (81%) with an age range of 26 to 60 years (mean age of 40). In this group, 61% were combat soldiers, 19% were combat support soldiers, and 20% were combat service support soldiers, with service periods ranging from 1 to 60 months (mean of 14 months). The reserve soldier group was older and predominantly male, reflecting typical demographic characteristics of the Israeli reserve system. Previous active-duty military service is clearly a prerequisite for reserve service, naturally making reserve service participants older. These differences are not incidental but reflect the real composition of reserve forces.

Note that in the IDF, soldiers are categorized based on their proximity to combat situations. Combat soldiers are trained to operate in direct combat zones and engage the enemy in close proximity, including roles such as infantry, armored corps, combat engineers, artillery, and special forces. Combat support soldiers provide essential services such as logistics, maintenance, intelligence, and medical support to combat units, often in or near combat zones. Combat service support soldiers serve in non-combat roles, typically administrative, technical, or logistical, and are based away from combat zones. These categorizations reflect the varying levels of risk and responsibility in each soldier’s role and may influence their PsyCap, social support, and SWB (see Table 1 for demographic information).

**Table 1:** Demographics for Active-Duty soldiers before and during the war and reserve soldiers during the war (*N* = 233)

Variables	Subscale	Active-duty soldiers before the war (*n* = 98)	Active-duty soldiers during the war (*n* = 76)	Reserve soldiers during the war (*n* = 59)
Gender	Males	58 (59%)	37 (49%)	48 (81%)
	Females	40 (41%)	39 (51%)	11 (19%)
Age	Age Range	18–27 (M = 21)	18–27 (M = 21)	26–60 (M = 40)
Service Type	Combat Soldier	39 (40%)	14 (18%)	36 (61%)
	Combat Support Soldier	31 (31%)	16 (21%)	11 (19%)
	Combat Service Support Soldier	28 (29%)	46 (61%)	12 (20%)
Service period	Months	10–80 (M = 22)	10–68 (M = 32)	1–60 (M = 14)

### Instruments

We used four questionnaires to test the research hypotheses.

### Demographic information

Demographic information was collected using a questionnaire designed for the study, gathering relevant data on participants’ age, gender, service type, and service duration. The questionnaire included questions about gender (male or female), age, and the type of military service performed. The service type categories were combat soldiers, combat support soldiers, and combat service support soldiers, each defined based on the soldier’s proximity to direct combat situations. The questionnaire also asked participants about the duration of their military service.

### PsyCap questionnaire

Participants’ PsyCap was determined using an academic shortened version of the PsyCap questionnaire, PCQ-24 [61], in which four positive psychological capacities (self-efficacy, hope, resilience, optimism) are applied to academic outcomes. The 12-item version of the PCQ-24 was validated by Avey, Avolio, and Luthans [62]. The PCQ-12 comprises 12 items rated on a 5-point Likert scale, from 1 = strongly disagree, to 5 = strongly agree. The score range of the PCQ is 12–60, with higher scores indicating higher levels of PsyCap. Table 2 shows the division of the subscales, including their reliability in this study.

**Table 2:** Division and reliability of PCQ subscales

Scales	α	Sample Item
Self-efficacy	0.76	I feel confident analysing a study-related long-term problem to find a solution.
Hope	0.79	There are lots of ways around any study-related problem.
Optimism	0.67	When things are uncertain for me as a student, I usually expect the best.
Resilience	0.60	I can deal with study-related difficulties because I’ve experienced difficulty before.
Overall	0.88	

### Social support questionnaire

The Multidimensional Scale of Perceived Social Support measures individuals’ social support according to their perceptions [63]. The questionnaire consists of 12 items examining the social support of family, friends, and other significant figures, ranked on a Likert scale from 1 = a situation not applicable to the respondent, to 7 = a situation highly applicable to the respondent. The average score of respondents’ answers is calculated; a higher score indicates higher social support. Table 3 shows the division of the subscales, including their reliability in this study.

**Table 3:** Division and reliability of social support subscales

Scales	α	Sample Item
Family	0.90	My family really tries to help me.
Friends	0.91	I have friends with whom I can share my joys.
Significant Other	0.85	There is a special person who is around when I am in need.
Overall	0.89	

### SWB questionnaire

SWB was assessed using the Subjective Well-Being Index (SWB-A) [64]. The SWB-A is composed of one question inquiring about satisfaction with life as a whole and eight items measuring satisfaction in specific life domains: standard of living, personal health, achieving in life, personal relationships, personal safety, community-connectedness, future security, and religion. All items are rated on a scale, ranging from 0 = completely dissatisfied, to 10 = completely satisfied. The internal consistency of the scale for this study was 0.90.

### Study procedure

Data were collected using digital questionnaires designed in Qualtrics software and randomly distributed to the participants. Prior to completing the questionnaires, all participants signed an informed consent form. The consent form provided a detailed explanation of the study’s objectives, procedures, and the voluntary nature of participation. Each participant responded to the questionnaires individually, and the average completion time was approximately 10 min. The instruction provided to the respondents was to answer the questions as honestly as possible, reflecting their feelings at the time of completing the questionnaire.

The research tools were administered in the following order: first, the Demographic Information Questionnaire, followed by the PsyCap Questionnaire, the SWB Questionnaire, and finally, the Social Support Questionnaire. All collected data were subsequently transferred to IBM SPSS Statistics version 28 for analysis.

The first phase of data collection involved active-duty soldiers during peacetime. The second phase of data collection occurred about a year later, approximately four months after the outbreak of the war in Gaza. This timing enabled the investigation of potential shifts in PsyCap, SWB, and social support in response to the demanding conditions of military service during conflict. Due to the high frequency of reserve soldier mobilizations, additional data were collected from a sample of reserve soldiers at the second time point. This allowed comparative analysis and yielded insights into the characteristics and functioning of each population group during IDF service (active-duty soldiers in peacetime, active-duty soldiers during war, and reserve soldiers during war), providing a comprehensive understanding of the impact of the military service context on the psychological and social factors measured in this study.

## Results

### Active-Duty soldiers: Pre-War and during war

Our first research question asked whether there would be positive correlations between PsyCap, social support, and SWB, both in general and within each of the active-duty groups: active-duty soldiers who participated in the study before the war and active-duty soldiers who participated during the war. We hypothesized significant positive correlations between variables both in the overall sample and within each group separately. The first hypothesis was partially corroborated: PsyCap was positively correlated to SWB in the entire sample and also for both groups. Social support was significantly and positively correlated to PsyCap and SWB in the entire sample and in the subsample of active-duty soldiers who participated in the study during the war (see Table 4).

**Table 4:** Means, standard deviations, and correlations between study variables (*N* = 174)

Variables	M (SD)	1	2
	**Total (** ***N*** ** = 174)**		
**1. PsyCap**	3.64 (0.64)		
**2. Social Support**	6.04 (0.85)	0.156*	
**3. SWB**	7.46 (1.46)	0.584***	0.306***
	**Pre-War (** ***n*** ** = 98)**		
**1. PsyCap**	3.60 (0.64)		
**2. Social Support**	6.16 (0.76)	0.097	
**3. SWB**	7.15 (1.36)	0.657***	0.183
	**During the War (** ***n*** ** = 76)**		
**1. PsyCap**	3.68 (0.63)		
**2. Social Support**	5.88 (0.94)	0.247*	
**3. SWB**	7.85 (1.50)	0.510***	0.525***

*p < .05 ** *p* < .01 *** *p* < .001

The second research question targeted possible differences between active-duty soldiers who participated in the study before the war and active-duty soldiers who participated in the study during the war in their levels of PsyCap, social support, and SWB. We hypothesized active-duty soldiers who participated in the study during the war would exhibit higher levels of social support and SWB than active-duty soldiers who participated in the study before the war, but both groups would have similar levels of PsyCap. Three *t*-tests fully confirmed this hypothesis (Table 5).

**Table 5:** Differences between Active-Duty soldiers during and before the war (*N* = 174)

Variables	Pre-War (*n* = 98)	During the War (*n* = 76)		
	M (SD)		t (172), *p*	Cohen’s d
**PsyCap**	3.60 (0.64)	3.68 (0.63)	0.754, *p* = .452	125.
**Social Support**	6.16 (0.76)	5.88 (0.94)	2.194, *p* < .05	0.408
**SWB**	7.15 (1.36)	7.85 (1.50)	3.171, *p* < .01	0.488

**Fig. 1:**
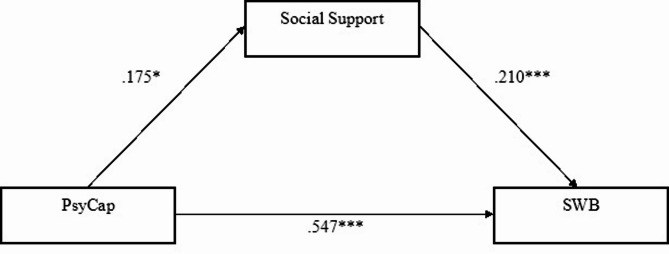
Relations between PsyCap and SWB via Social Support as a Mediator: Active-Duty Soldiers Before and During the War

Our third research question asked whether social support would mediate the relations between PsyCap and SWB among all active-duty soldiers. We hypothesized higher levels of PsyCap would be associated with higher levels of social support, which, in turn, would lead to greater SWB. We used a PROCESS procedure to examine this hypothesis, calculating PsyCap as the independent variable, SWB as the dependent variable, and social support as a mediator. Findings showed a statistically significant, positive, and direct relationship between PsyCap and social support, B = 0.230, S.E.=0.099, CI; 0.034–0.426, and between social support and SWB, B = 0.365, S.E.=0.106, CI; 0.155–0.575. The relationship between PsyCap and SWB was also statistically significant, B = 1.256, S.E.=0.140, CI; 0.979–1.533 (see Fig. 1).

To test the significance of the indirect effect in the analysis, we employed a bootstrapping technique, using 5,000 resamples, to generate 95% confidence intervals (CIs). Indirect effects in which zero is not included in the 95% CI indicate a significant effect at α < 0.05. Tests of the indirect effect of PsyCap on SWB via social support showed this was significant, B = 0.084, S.E.= 0.046, CI; 0.012–0.193.

Our fourth research question asked whether there would be positive correlations between PsyCap, social support, and SWB, among all soldiers serving in the Gaza war and within two subgroups: active-duty soldiers and reserve service soldiers. We hypothesized significant positive correlations both in the overall sample and within each of the groups separately. This hypothesis was fully corroborated (see Table 6).

**Table 6:** Means, standard deviations, and correlations between study variables (*N* = 135)

Variables	M (SD)	1	2
	**Total (** ***N*** ** = 135)**		
**1. PsyCap**	3.85 (0.64)		
**2. Social Support**	5.94 (0.88)	0.323***	
**3. SWB**	8.14 (1.50)	0.564***	0.542***
	**Active Duty (** ***n*** ** = 89)**		
**1. PsyCap**	3.68 (0.63)		
**2. Social Support**	5.88 (0.94)	0.247*	
**3. SWB**	7.85 (1.50)	0.510***	0.525***
	**Reserve Service (** ***n*** ** = 59)**		
**1. PsyCap**	4.07 (0.58)		
**2. Social Support**	6.00 (0.79)	0.437***	
**3. SWB**	8.52 (1.42)	0.569***	0.575***

*p < .05 ** *p* < .01 *** *p* < .001

The fifth research question targeted possible differences between active-duty soldiers and reserve service soldiers serving in the Gaza war in their levels of PsyCap, social support, and SWB. We hypothesized reserve service soldiers would have higher levels of all three variables than active-duty soldiers. Three *t*-tests partially confirmed this hypothesis, indicating differences between groups in PsyCap and SWB, but not social support (Table 7).

**Table 7:** Differences between Active-Duty soldiers and reserve service soldiers (*N* = 135)

Variables	Active Duty (*n* = 89)	Reserve Service (*n* = 59)		
	M (SD)		t (133), *p*	Cohen’s d
**PsyCap**	3.68 (0.63)	4.07 (0.58)	3.725, *p* < .001	644.
**Social Support**	5.88 (0.94)	6.00 (0.79)	0.774, *p* = .441	0.138
**SWB**	7.85 (1.50)	8.52 (1.42)	2.624, *p* < .01	0.458

**Fig. 2:**
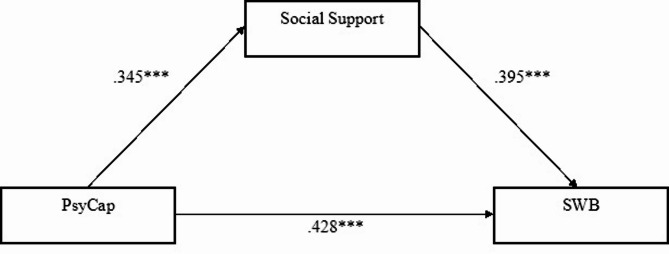
Relations between PsyCap and SWB via Social Support as a Mediator: Active-Duty vs. Reserve Service Soldiers

Our sixth and final research question asked whether social support would mediate the relations between PsyCap and SWB among both active-duty soldiers and reserve service soldiers during the war. We hypothesized higher levels of PsyCap would be associated with higher levels of social support in both groups, and this, in turn, would lead to greater SWB. A PROCESS procedure was used to examine this hypothesis, calculating PsyCap as the independent variable, SWB as the dependent variable, and social support as a mediator. Findings showed a statistically significant, positive, and direct relationship between PsyCap and social support, B = 0.468, S.E.=0.111, CI; 0.248–0.687, and between social support and SWB, B = 0.683, S.E.=0.119, CI; 0.446–0.918. PsyCap and SWB also demonstrated statistically significant correlations, B = 1.002, S.E.=0.162, CI; 0.683–1.322 (see Fig. 2). Tests of the indirect effect of PsyCap on SWB via social support showed this was significant, B = 0.319, S.E.= 0.098, CI; 0.149–0.541.

## Discussion

The study explored the relationships between PsyCap, social support, and SWB in three samples of IDF soldiers: active-duty soldiers serving before the Gaza war, active-duty soldiers serving in the Gaza war, and reserve service soldiers serving in the Gaza war. The findings provide valuable insights into the role of psychological resources and external support in shaping soldiers’ well-being, especially in conflict. Findings confirmed positive correlations between PsyCap, social support, and SWB, with social support mediating this relationship. Differences emerged between active-duty soldiers serving before and during the war, as well as between active-duty and reserve service soldiers serving during the war, underscoring the impact of the military context on well-being.

H1 proposed PsyCap, social support, and SWB would be positively correlated across the whole sample of active-duty soldiers and within the samples of active-duty soldiers serving before and during the war. H1 was partially confirmed. We found significant positive correlations between PsyCap and SWB in the total sample and within each group separately, reinforcing the notion that PsyCap contributes to well-being in military settings. Social support was positively correlated with both PsyCap and SWB; however, this relationship was significant only in the overall sample and among active-duty soldiers during the war, not in the pre-war group.

Studies have highlighted the negative psychological effects of combat experience on soldiers’ well-being [43, 44]. Exposure to combat has been linked to declines in SWB, with soldiers frequently facing long-term psychological challenges such as PTSD and depression [7, 32, 45]. However, strong interpersonal relationships can serve as a protective buffer against these adverse effects. Soldiers with close bonds, whether with intimate partners or fellow service members, tend to report higher levels of SWB, underscoring the crucial role of social support in mitigating the psychological toll of military service [10, 45]. Our findings suggest that while in routine situations, soldiers may not rely solely on social support to enhance their psychological resources and sense of personal well-being, likely drawing on additional anchors, during wartime, reliance on personal psychological factors and social support becomes more significant. However, given the non-experimental design, such conclusions must be interpreted with caution, as we cannot determine temporal or causal directionality.

H2 argued there would be differences between active-duty soldiers who participated in the study before the war and those who participated during the war in their levels of PsyCap, social support, and SWB. Specifically, while PsyCap levels would remain stable across both groups, active-duty soldiers who participated during the war would report higher levels of social support and SWB. The results confirmed this hypothesis. The stability of PsyCap levels across pre-war and wartime conditions suggests that while PsyCap is a significant internal resource, it may not fluctuate dramatically over short periods, even in response to heightened stress. Instead, the increase in social support and SWB among soldiers during the war may reflect the critical role of shared purpose and unit cohesion. In combat situations, strong interpersonal bonds and a sense of collective mission can enhance social support, which in turn, bolsters psychological well-being [39, 65]. Previous studies have suggested social support acts as a protective buffer against stress, particularly in high-risk occupations like military service. Soldiers who perceive strong connections with their peers tend to show greater psychological resilience, which contributes to enhanced well-being [10]. Furthermore, the experience of working toward a common goal, such as protecting one’s country and ensuring the survival of fellow soldiers, can strengthen a sense of meaning and purpose, leading to improved SWB. This is consistent with research showing purpose-driven environments contribute positively to emotional and psychological resilience [45].

H3 argued social support would mediate the relationship between PsyCap and SWB among all active-duty soldiers, those who participated in the study before the war and those who participated during the war. We hypothesized higher levels of PsyCap would be associated with greater social support, which in turn, would enhance SWB. The findings confirmed this hypothesis, revealing statistically significant, positive correlations between PsyCap and social support, social support and SWB, and a direct relationship between PsyCap and SWB. These results align with literature underscoring the critical role of social support in well-being, particularly in high-stress environments like military service [9, 49]. Social support is widely recognized as a protective factor that mitigates stress by reinforcing feelings of being valued, cared for, and connected to others. It consists of multiple dimensions, including emotional support (providing reassurance and validation), tangible support (such as financial or logistical aid), and informational support (guidance and problem-solving assistance) [50]. The role of social support is particularly pronounced in military contexts, where soldiers undergo a transition from civilian life to a regimented and demanding environment. The enlistment and recruitment process in Israel is mandatory and often introduces significant psychological strain, as individuals adapt to strict hierarchies, combat training, and high-pressure decision-making [55]. During this period, social support within military units can serve as a critical buffer against psychological distress, fostering a sense of belonging and reducing the likelihood of negative mental health outcomes such as depression or substance abuse [10].

Our finding of the mediating effect of social support in the relationship between PsyCap and SWB aligns with previous findings showing resilience, optimism, hope, and self-efficacy, key components of PsyCap, can reinforce strong social networks [36]. Soldiers with higher PsyCap may be more likely to seek and provide support within their units, thereby strengthening their psychological resilience. The shared experience of military service fosters unit cohesion, which facilitates practical assistance and also reinforces the psychological resources necessary to maintain well-being under stress. Furthermore, social support has been found to reduce the impact of trauma-related psychopathology, emphasizing its role in sustaining mental health in military settings [18].

Beyond immediate psychological benefits, the buffering role of social support has long-term implications for soldiers’ well-being. Soldiers who perceive strong social support are more likely to develop adaptive coping mechanisms, exhibit higher self-efficacy, and maintain a positive outlook despite the challenges of military service. These factors, in turn, contribute to sustained well-being and may even promote post-traumatic growth, allowing soldiers to find meaning and resilience in adversity.

H4 proposed PsyCap, social support, and SWB would be positively correlated among active-duty and reserve soldiers serving during the Gaza war, both in the overall sample and within the two groups. H4 was confirmed. Others have similarly found the components of PsyCap serve as protective factors for soldiers in high-stress environments, such as combat [31, 35]. Soldiers with higher levels of PsyCap are more likely to experience enhanced resilience and improved well-being [6, 33]. Moreover, social support has been found to play a pivotal role in buffering against the negative effects of stress, including the psychological toll of combat [10, 45]. As in previous studies, soldiers in our sample with strong interpersonal relationships, particularly family support, reported higher levels of SWB and lower rates of psychological distress post-service [45, 53]. This interplay between PsyCap and social support strengthens the capacity of soldiers to cope with military challenges and contributes positively to their SWB [11, 19].

H5 argued reserve service soldiers would have higher levels of PsyCap, social support, and SWB than active-duty soldiers. H5 was partially supported. There were significant differences between groups in their levels of PsyCap and SWB but no significant differences in social support. Despite the expectation that reserve service soldiers might have higher perceived social support, both groups reported similarly strong levels, with no statistically significant differences.

This finding suggests the foundation of social support among military personnel is largely established during active-duty service, when soldiers undergo shared hardships, face life-threatening situations, and operate under a collective mission-oriented mindset [66]. In the Israeli case, all reserve soldiers have been active-duty service members at some point. Thus, the social bonds formed during their time in active service persist even after transitioning to civilian life. These bonds can be renewed and reinforced during times of national crisis when reserve soldiers are called back into service, reactivating the sense of camaraderie and shared purpose forged in their earlier military experiences [67]. Additionally, the military structure itself fosters strong unit cohesion, a key determinant of perceived social support, which does not necessarily diminish when a soldier transitions into reserve service [68]. Another important aspect is the psychological impact of collective resilience during wartime or military operations. Soldiers, both active-duty and reserve, rally around a common goal, strengthening interpersonal support networks. In times of conflict, shared experiences and mutual reliance on comrades provide an enduring source of emotional and instrumental support, explaining why both groups reported equally strong levels of social support [69].

We found significant differences in the groups in their levels of PsyCap and SWB, with reserve service soldiers showing higher levels of both than active-duty soldiers. This result aligns with previous research suggesting life experiences, career development, and personal responsibilities play a crucial role in shaping psychological resilience and overall well-being [36]. The increased PsyCap in reserve service soldiers may stem from their transition into civilian life, where they navigate complex challenges such as career development, financial independence, and family responsibilities [70]. These experiences may foster a greater sense of self-efficacy and problem-solving skills, contributing to higher PsyCap levels.

Similarly, SWB was significantly higher among reserve service soldiers than active-duty soldiers. One possible explanation is that reserve soldiers, having completed their mandatory service, have had the opportunity to establish personal and professional stability. Many have pursued higher education, career advancement, and family life, factors that contribute positively to life satisfaction and emotional well-being [71]. In contrast, active-duty soldiers are still embedded within the demanding and unpredictable structure of military life, thus limiting their sense of autonomy and control over personal life decisions and affecting their overall well-being [72].

H6 posited social support would mediate the relationship between PsyCap and SWB in both active-duty soldiers and reserve service soldiers serving in the Gaza war. The findings showed higher PsyCap was positively associated with social support, which, in turn, enhanced SWB, thus supporting the role of social support as a key mechanism linking PsyCap to well-being. Other studies have similarly found PsyCap fosters positive social interactions and prosocial behaviors, strengthening support networks [33, 36]. Individuals with high PsyCap communicate their needs effectively and engage in reciprocal support, reinforcing social ties [21, 73] and, in turn, experiencing better SWB. This is especially relevant in military contexts, where unit cohesion and camaraderie are vital for psychological resilience.

### Theoretical contribution

Although PsyCap and SWB are well-established constructs [36, 74], our study contributes novel insights in three primary ways. First, we apply these constructs to a highly specific and understudied population: Israeli soldiers in a real-time war zone (the 2023 Gaza conflict). Most prior studies explore PsyCap in civilian or peacetime military settings [33, 34], whereas our study captures dynamic psychological responses to imminent life threat. Second, we empirically test social support as a mediating mechanism in the relationship between PsyCap and SWB under varying service conditions (active duty vs. reserve). This relational model builds on and extends COR theory [21] by demonstrating how internal psychological resources catalyze external social ones, creating a resilience-building loop. Third, our comparative analysis of reserve and active-duty soldiers reveals how life-course experiences shape PsyCap, suggesting it may not only be a trait-like resource but also be molded by civilian reintegration and life challenges [70]. These findings advance theory by suggesting the contextual malleability of PsyCap in post-military trajectories.

### Limitations and future research

While this study provides significant insights into the role of PsyCap and social support in fostering well-being in the military, several areas warrant further exploration. One of the limitations is the cross-sectional nature of the research design, which does not allow the examination of the causal relationships between PsyCap, social support, and SWB over time. Future research should adopt longitudinal designs to examine how they evolve, particularly in response to prolonged combat exposure and post-service reintegration. Moreover, although the findings suggest reserve soldiers exhibit higher levels of PsyCap, potentially due to life experience, civilian reintegration, or age, the cross-sectional design does not permit causal or developmental conclusions. We cannot determine whether PsyCap was shaped over time or predated military experiences. Future longitudinal studies are needed to examine how PsyCap evolves in response to life transitions, particularly the shift between civilian and military roles.

Another limitation is the reliance on self-reported data, which may be subject to biases such as social desirability or recall bias. To mitigate this limitation, experimental studies assessing the effectiveness of PsyCap and social support interventions within military settings can provide empirical validation for targeted psychological training programs.

A further limitation is that we sampled only IDF soldiers, restricting the generalizability of the findings. Investigating these variables across different military forces and cultural contexts can offer a broader understanding of how psychological resilience operates in diverse geopolitical environments.

Finally, while we considered controlling age and gender statistically, we chose not to do so because these characteristics are inherently tied to group identity (i.e., reserve vs. active-duty). We now justify this decision explicitly. While the study uses cross-sectional, self-report data, it includes two distinct data waves collected approximately one year apart before and during war, thus approximating a quasi-longitudinal design. This provides insight into temporal change, a strength rarely present in wartime studies [43]. Future research with larger samples should consider using a moderated mediation approach (e.g., PROCESS Model 59) to formally test whether the mediating role of social support differs by type of military service.

## Conclusions

Findings showed significant positive correlations between PsyCap, social support, and SWB among soldiers, with social support mediating the relationship. While PsyCap remained stable across different conditions (pre-war, during war) among active-duty soldiers, social support and SWB were more pronounced among soldiers serving during wartime, highlighting the crucial role of interpersonal connections in high-stress environments. The results reinforce the theoretical assumption of an association between PsyCap, social support, and well-being, particularly in military contexts. Additionally, the mediation effect of social support suggests the importance of social networks in mitigating the adverse effects of combat stress and improving SWB. However, conclusions about directionality or causality must remain tentative due to the study’s correlational nature.

The results we found for the different types of military service merit further attention. Notably, reserve soldiers had higher levels of PsyCap and SWB than active-duty soldiers. This finding suggests the potential for these soldiers to assist regular soldiers in these domains, thereby maximizing the likelihood that they, too, will experience enhanced levels of PsyCap – a resource that can be modified and developed – as well as higher perceptions of social support and SWB.

Beyond empirical findings, this study contributes to theory by applying PsyCap, SWB, and social support constructs in a combat zone, expanding the boundaries of their applicability. The integrated mediation model illustrates how personal psychological strengths and interpersonal resources combine to support well-being during extreme adversity, offering a contextual extension to COR theory and advancing models of military resilience.

### Recommendations and suggestions for policymakers

The findings have practical implications for military organizations, policymakers, and mental health professionals. Military institutions could integrate PsyCap development programs into their training curricula, as these resources can be developed through structured interventions [58]. Given the importance of PsyCap and social support in promoting SWB, military organizations could implement structured peer support programs, mentorship initiatives, and unit cohesion activities. Strengthening social bonds within military units may significantly improve soldiers’ emotional resilience and adaptive coping strategies, ultimately enhancing their overall well-being. Our findings highlight the importance of peer-based emotional support and unit cohesion in promoting well-being during wartime. Policy efforts should prioritize strengthening these ties through team-building and peer-support activities. Finally, reserve soldiers who show higher PsyCap and SWB could be integrated into mentoring roles to support active-duty personnel with both emotional resilience and practical guidance.

## Data Availability

No datasets were generated or analysed during the current study.
